# Influence of sports compression textiles on endurance running performance

**DOI:** 10.1186/2046-7648-4-S1-A86

**Published:** 2015-09-14

**Authors:** Martin Harnisch, Anke Klepser, Edith Classen, Jan Beringer, Andreas Schmidt

**Affiliations:** 1 Hohenstein Institut fuer Textilinnovation gGmbH, Boennigheim, Germany

## Introduction

Sports compression textiles are very popular, especially in endurance sports like triathlon and marathon running [1]. It is well known, that medical compression stockings reduce the expansion of veins and a compressive gradient to the heart enhances venous blood flow [1-3]. During sports compressive textiles reduce wobbling masses, muscle vibration and swelling [4]. Therefore compression should lead to better endurance performance, but the benefit on endurance performance is not clearly proved, e.g. Kemmler *et al*. [5] found a benefit by compression textiles while Sperlich *et al*. [1] did not.

## Methods

Four compression stockings/calf sleeves with different compression and compression gradients as well a combination of compression stocking with compression thigh sleeve were tested against short sleeved sportswear without compression. Ten experienced runners (44.6 ± 4.5 years) participated in running trials on a treadmill in a climatic room (T_a _= 23 ± 0.2 °C, RH_a _= 56 ± 6% rh). An incremental test was performed to find individual running speed. The main test included 40 min at 70% of maximum oxygen consumption followed by 5 min at speed of penultimate increment. Arterial lactate concentration, oxygen consumption, heart rate, core temperature, skin temperature and humidity and weight loss were recorded. Subjects gave feedback on temperature, humidity, compression, exertion and general perception.

## Results

Statistical differences could not be found for heart rate, oxygen consumption and lactate concentration when comparing different compression textiles and common sportswear. Core temperature, mean whole body temperature and humidity were not increased when wearing compression textiles in comparison to common short sleeved sportswear. But temperature and humidity at calf and thigh were higher when covered by compression textiles. Furthermore weight/sweat loss was not significantly higher when wearing compression textiles and efficiency of evaporation (ratio of produced to evaporated sweat) was not affected. Perception of temperature and humidity was not worse in comparison to common sportswear, but compression at legs made subjects feel better and less exhausted.

## Discussion

Compression textiles had no influence on physiological parameters describing endurance performance, e.g. heart rate, oxygen consumption and lactate, what corresponds with findings by Sperlich *et al*. [1]. Furthermore it was found that this is independent of compression and compression gradient provided by stockings or sleeves for calve and thigh. On the other hand it could be shown, that an additional clothing layer by compression textiles did not lead to higher thermal stress. In addition subjects felt less exhausted when wearing compression textiles, at the end of the series a ranking of all clothing systems showed a preference for compression textiles by subjects in comparison to standard sportswear.

## Conclusion

There is no physiological evidence for a benefit of compression textiles on running endurance. On the other hand compression textiles do not add thermal stress, show a psychological benefit and compression makes subjects feel less exhausted.
